# Cost Utility of Specialist Physiotherapy for Functional Motor Disorder (Physio4FMD): Economic Analysis of a Pragmatic Randomized Controlled Trial

**DOI:** 10.1212/CPJ.0000000000200465

**Published:** 2025-04-01

**Authors:** Rachael Maree Hunter, Glenn Nielsen, Marie Le Novere, Louise Marston, Teresa C. Lee, Jon Stone, Laura H. Goldstein, Alan Carson, Kate Holt, Jonathan Marsden, Irwin Nazareth, Hayley Noble, Markus Reuber, Ann-Marie Strudwick, Beatriz Santana Suarez, Mark J. Edwards

**Affiliations:** 1 Department of Primary Care and Population Health, University College London, United Kingdom;; 2 PRIMENT Clinical Trials Unit, University College London, United Kingdom;; 3 Neuroscience Research Centre, Institute of Molecular and Clinical Sciences, St. George's University of London, United Kingdom;; 4 Clarivate;; 5 Department of Statistical Science, University College London, United Kingdom;; 6 Centre for Clinical Brain Sciences, Royal Infirmary of Edinburgh, United Kingdom;; 7 Department of Psychology, Institute of Psychiatry, Psychology and Neuroscience, King's College London, United Kingdom;; 8 Faculty of Health, School of Health Professions, University of Plymouth, United Kingdom;; 9 Academic Neurology Unit, University of Sheffield, United Kingdom;; 10 Neuroscience, Research and Innovation, King's College Hospital, London, United Kingdom;; 11 Department of Basic and Clinical Neuroscience, Institute of Psychiatry, Psychology and Neuroscience, London, United Kingdom; and; 12 Department of Neuropsychiatry, Maudsley Hospital, London, United Kingdom.

## Abstract

**Background and Objectives:**

Functional motor disorder (FMD), a motor-dominant variant of functional neurologic disorder, is a disabling condition associated with high health and social care resource use and poor employment outcomes. Specialist physiotherapy presents a possible treatment option, but there is limited evidence for clinical effectiveness and cost-effectiveness. Physio4FMD is a multicenter randomized controlled trial of specialist physiotherapy for FMD compared with treatment as usual (TAU). The aim of the analysis was to conduct a randomized trial based on economic evaluation of specialist physiotherapy compared with TAU.

**Methods:**

Eleven centers in England and Scotland randomized participants 1:1 to specialist physiotherapy or TAU (referral to community neurologic physiotherapy). Participants completed the EuroQoL EQ-5D-5L, Client Service Receipt Inventory, and Work Productivity and Activity Impairment Questionnaire at baseline, 6 months, and 12 months. The mean incremental cost per quality-adjusted life year (QALY) for specialist physiotherapy compared with TAU over 12 months was calculated from a health and social care and wider societal perspective. The probability of cost-effectiveness and 95% CIs were calculated using bootstrapping.

**Results:**

The analysis included 247 participants (n = 141 for specialist physiotherapy, n = 106 for TAU). The mean cost per participant for specialist physiotherapy was £646 (SD 72) compared with £272 (SD 374) for TAU. Including the costs of treatment, the adjusted mean health and social care cost per participant at 12 months for specialist physiotherapy was £3,814 (95% CI £3,194–£4,433) compared with £3,670 (95% CI £2,931–£4,410) for TAU, with a mean incremental cost of £143 (95% CI £–825 to £1,112). There was no significant difference in QALYs over the 12-month duration of the trial (0.030, 95% CI –0.007 to 0.067). The mean incremental cost per QALY was £4,133 with an 86% probability of being cost-effective at a £20,000 threshold. When broader societal costs such as loss of productivity were taken into consideration, specialist physiotherapy was dominant (incremental cost: £−5,169, 95% CI £–15,394 to £5,056).

**Discussion:**

FMD was associated with high health and social care costs. There is a high probability that specialist physiotherapy is cost-effective compared with TAU particularly when wider societal costs are taken into account.

**Trial Registration Information:**

International Standard Randomised Controlled Trial registry, ISRCTN56136713.

## Introduction

Functional neurologic disorder (FND) is a common cause of neurologic symptoms (such as weakness and sensory deficits of seizures) and disability in neurologic practice.^1^ Functional motor disorder (FMD) describes a subset of people with FND who have movement symptoms such as limb weakness, tremor, or walking problems with impaired voluntary control and perception of their bodies.^2^ FND represents a significant cost to health systems, patients, and their families, with an annual cost per patient in 2021 estimated between $21,433 and $86,722 in US Dollars, with the variation occurring by data collection method and country, with most of the studies from the United States. Indirect costs such as productivity loss and impact on carers make up two thirds of that cost.^3^ The process of navigating the health care system to obtain a diagnosis for FND can be lengthy and costly, with an Italian study finding that it could take over 6 years and cost 2,302 Euros per patient on average.^4^ Earlier diagnosis can be associated with a reduced cost per patient to the health care system.^3^

There is currently limited evidence for cost-effective interventions in FND, with a systematic review published in 2023 (3) identifying only two economic evaluations of FND trials alongside randomized control trials, with only 1 calculating an incremental cost per quality-adjusted life year (QALY) gained that met the best practice guidance for conducting economic evaluations.^5^ The review also found limited evidence for the validation of measures used to collect health care resource use and poor measurement of the impact on costs outside of health and social care.^3^

Although there is limited evidence for effective treatments of FND, specialist physiotherapy has shown some promise.^6^ We developed a specialist physiotherapy protocol for FMD. The intervention is protocolized to be individually tailored to specific needs and aims to target unhelpful expectations and reduce excessive self-directed attention. A feasibility RCT of the intervention found that it was cost-effective at 6-month follow-up, with an incremental cost per QALY gained of £12,087 and with promising clinical outcomes, with 72% of the participants in the specialist physiotherapy group rating their symptoms as improved on a 5-point scale, compared with 18% receiving treatment as usual (TAU).^5^

Physio4FMD was an RCT to evaluate the clinical effectiveness and cost-effectiveness of a specialist physiotherapy intervention for FMD compared with TAU, with the primary clinical outcome being the Physical Functioning domain of the SF36 at 12 months after randomization. The primary aim of this article was to report the mean incremental cost per QALY gained with specialist physiotherapy compared with referral to a community physiotherapist (TAU) for people with FMD using participant-level RCT data over 12 months.

## Methods

Reporting for the study is in compliance with the Consolidated Health Economic Evaluation Reporting Standards 2022, and a copy of the complete checklist is available in eSAP 1.

### Study Design and Participants

Eleven investigation centers in England and Scotland recruited patients with a diagnosis of FMD from outpatient neurology clinics and inpatients to a pragmatic, multicenter, parallel-group randomized controlled trial. Participants were eligible for recruitment if (1) they were new or returning patients; (2) they had a “clinically definite” diagnosis of FMD according to the Gupta and Lang diagnostic classification criteria^7^; (3) they were aged 18 years or older; (4) their diagnostic investigations had come to an end; (5) the patient was accepting of the intervention; and (6) the motor symptoms caused significant distress or impairment in social, occupational, or other important areas of functioning (subjectively described by the patient), independent of other comorbidities. Patients were excluded from the trial if they had severe comorbidities that would interfere with their ability to undertake physiotherapy, including severe pain or fatigue that would prevent them from engaging in physiotherapy, had disability to the extent that they require assistance for toileting, are unable to complete the questionnaires including unable to sufficiently understand English or had a documented learning disability. Ongoing unresolved compensation claims or litigation and the patient having no fixed address or was seeking rehousing were also grounds for exclusion. Patients needed to have the capacity to give consent to take part in the trial. Further information on trial design can be found in the protocol,^8^ analysis plan,^9^ and main clinical trial article.^10^ All participants had to provide written informed consent.

### Randomization and Masking

After completing baseline assessment, participants were randomized (1:1) to specialist physiotherapy or TAU (referral to community neurologic physiotherapy) by the trial manager at St George's University of London. Block randomization with random block sizes was used to ensure even allocation between randomized groups, stratified by site. This was conducted using a web-based application created by an independent company, “Sealed Envelope.”^11^ Researchers collecting the trial outcomes, statisticians, and health economists were masked to treatment allocation, and participants were asked not to reveal their group allocation to researchers. Owing to the nature of the intervention, it was not possible to mask the trial manager, participants, or treating clinicians.

### Procedures

#### Specialist Physiotherapy

Physiotherapists specializing in neurorehabilitation delivered the intervention in line with a protocolized physiotherapy program, which could be adapted to the needs of individuals. The program consisted of 9 sessions delivered within a three-week period, plus a single follow-up session after 3 months. Physiotherapy sessions were guided by an interactive workbook, which formed part of the self-management plan. The treatment has been described in more detail elsewhere.^8^ As part of the trial, physiotherapists completed a 5-day training program before delivering treatment.

TAU represents referral to community neurologic physiotherapy. The comparator condition was TAU, defined as a referral made by the diagnosing neurologist to the National Health Service (NHS) community neurologic physiotherapy service.

### Cost of the Intervention

The cost of the specialist physiotherapy intervention included the time for the physiotherapist to deliver the intervention, plus the cost of training and supervision of the therapist by a senior physiotherapist. Treatment as usual included the cost of the physiotherapy sessions only. The intervention was costed as being delivered by a hospital-based physiotherapist of seniority levels similar to those in the study and the cost of training by a more senior physiotherapist.

### Resource Use and Costs

Resource use in both groups was collected from self-reported questionnaires at baseline, 6 months, and 12 months asking about the previous 6 months.

Health and social care resource use was collected using a version of the Client Service Receipt Inventory (CSRI) adapted based on the experience of the feasibility study.^5^ The CSRI asked about general practitioner contacts, social care, physiotherapy other than that received as part of the trial, other community health care, specialist medical outpatient appointments, hospital attendances, and home adaptations. The CSRI also collected information on welfare payments, months of full-time or part-time employment, Office of National Statistics work category to calculate mean pay (with different pay for full-time or part-time work), and days of sick leave.^12^

The unit costs for costing resource use from the CSRI are reported in eTable 1. Paid and unpaid carer time was calculated using the Institute for Medical Technology Assessment Valuation of Informal Care Questionnaire^13^ adapted based on our experience in other trials.^14^ Unpaid carer time was costed using the replacement cost method, assuming that it could be provided by a social home care worker.^15^

The Work Productivity and Activity Impairment (WPAI)^16^ questionnaire, which asks about the number of hours of work missed because of ill health in the past 7 days and degree that health affected productivity while working, was used to calculate presenteeism and absenteeism, where presenteeism is the lost productivity from illness while working and absenteeism is time off work while employed. Total potential earnings over 6 months were calculated based on the number of hours per week worked (37.5 hours per week assumed for full time and hours per week reported for part time), multiplied by the number of weeks worked. Absenteeism was calculated as the number of hours lost from work in the past week divided by the total number of hours that could have possibly been worked. Presenteeism was calculated from the question asking about how the participant's productivity while at work has been affected by their illness in the past 7 days divided by 10, the maximum value of the visual analog scale. Lost productivity and sick leave were costed based on the human capital approach and information reported in the CSRI and WPAI.

Follow-up assessments were conducted remotely using the participants' preferred method, by either an online form, return-mail paper forms, or telephone.

Additional health care use data were obtained from NHS England and NHS Scotland on accident and emergency (A and E) attendances, hospital-based appointments, and admissions. We removed any inpatient contacts that were maternity related from the data set. The data from NHS England, hospital episode statistics (HES), were costed using NHS Reference Costs.^17^ For 19 of a total of 797 inpatient attendances (2%), no unit cost could be identified. Resource use from 1 site was dropped because resources related to the trial were recorded in the HES data. Results from the HES analysis were compared with the results for the relevant costs for the CSRI to determine the validity of the CSRI costs. The Scottish data did not contain sufficient detail to allow for a full costing and hence were not used in this analysis.

All costs are reported in British pounds (GBP) and for the year 2021/2022.

### Outcome Measures

The EQ-5D-5L scores ^18^ were collected at baseline, 6 months, and 12 months to allow the calculation of QALYs. For the main analysis, EQ-5D-5L scores were converted to utility scores using the value set for England (VSE).^19^ A secondary analysis was conducted using the algorithm to map EQ-5D-3L scores to EQ-5D-5L scores.^20^

### Statistical Analysis

A combined statistics and health economics analysis plan was signed off and published before database lock.^9^

We calculated complete case (participants who were followed up at that time point and completed that section of the questionnaire) descriptive statistics for the percentage of participants and the mean number of contacts for each type of resource use. Complete case means and SDs for costs were also calculated. The mean difference in costs, 95% CI, and *p* value for each resource use type were calculated using regression analysis adjusting for baseline costs, with site as a covariate and accounting for therapist clustering as a random effect calculated using bias-corrected bootstrapping with 5,000 iterations for complete cases (available at all time points).

QALYs were calculated as the area under the curve using responses to the EQ-5D-5L.^21^ People who died before they reached a specific follow-up point are included as 0 for each follow-up point after they died, assuming a straight line from their last complete questionnaire until death. For the EQ-5D-5L, we report the mean values at each time point and mean unadjusted QALYs from baseline to 12 months. Mean difference in QALYs, 95% CI ,and *p* value were calculated using regression analysis adjusting for baseline utility,^21^ with site as a covariate and accounting for therapist clustering as a random effect calculated using bias-corrected 5,000 iterations for complete cases (available at all time points).

To ensure the inclusion of random-effect clustering for therapists, we used two-stage bootstrapping to calculate incremental costs and QALYs and construct cost-effectiveness planes and cost-effectiveness acceptability curves (CEACs).^22^ Because the method is nonparametric, it does not require distributional assumptions to be made about costs and outcomes. The only predictor of missingness was site, which is included in the regression analysis.

Some patients recruited to the trial directly before the COVID-19 pandemic and randomized to the intervention were unable to receive either all or part of the intervention, given its face-to-face nature. These patients were excluded from the analysis, as defined in the published analysis plan.^9^ Additional participants were recruited after the COVID-19 pandemic.

Because the trial-based analysis covers a 12-month duration, no discount rate was applied. Analyses were conducted using Stata version 17.^23^

### Perspective

For the main analysis, costs from a health and social care perspective only are reported. A secondary analysis covers wider societal costs including private health and social care, out-of-pocket costs, unpaid carer time, and the cost impact of presenteeism and absenteeism. The inclusions of welfare payments in economic evaluations are controversial, given that they can represent a transfer payment (there is not a net loss to the system because the money is transferred to another payer),^24^ and hence, total wider costs have been reported including and excluding welfare payments.

#### COVID-19 Subgroup Analyses

The primary cost-effectiveness analysis (health and social care perspective and using the VSE for QALYs) was run for patients recruited at different stages in the pandemic.^9^

### Standard Protocol Approvals, Registrations, and Patient Consents

The study was approved by the London-Surrey Borders Research Ethics Committee, under reference number 18/LO/0486, 28 March 2018. The trial was registered with the International Standard Randomised Controlled Trial registry, ISRCTN56136713.

### Data Availability

Deidentified participant data can be made available by request to the corresponding author. Requests will be considered after planned analyses and reporting have been completed by the investigators. Access will require submission of a protocol that is approved by a review committee and a signed data access agreement. Owing to the data-sharing agreement and for patient confidentiality reasons, we are not able to provide access to HES data from NHS England and NHS Scotland.

## Results

Between 19 October 2018–11 March 2020 and 3 August 2021–31 January 2022, with a 17-month break during the COVID-19 pandemic, 355 participants were randomized to specialist physiotherapy (n = 179) or TAU (n = 176). The primary analysis included only participants whose intervention was potentially unaffected by the COVID-19 period, with a total of 247 participants randomized (n = 141 in specialist physiotherapy, n = 106 in TAU), of whom 218 had completed CSRI at 6 months and 12 months (n = 128 in specialist physiotherapy, n = 90 in TAU) and 226 had completed EQ-5D-5L to calculate QALYs (n = 133 in specialist physiotherapy, n = 93 in TAU) (eFigure 1). Descriptive statistics for the included participants are presented in Table 1. Further details on the different COVID-19 groups and how they were determined are reported in our analysis plan and clinical effectiveness article.^8,25^

**Table 1:** Baseline Characteristics

	Specialist physiotherapy (n = 141)	Treatment as usual (n = 106)
Age, y		
Mean (SD)	45.0 (14.3)	44.4 (14.9)
Sex (%)		
Female	104 (73.8)	79 (74.5)
Ethnicity (%)		
Asian	6 (4.3)	2 (1.9)
Black	6 (4.3)	1 (0.9)
White	126 (89.4)	97 (91.5)
Mixed	2 (1.4)	5 (4.7)
Other	1 (0.7)	1 (0.9)
Relationship status (%)		
Married or cohabitating with partner	77 (54.6)	63 (59.4)
Single, separated, or widowed	64 (45.4)	43 (40.6)
Dependents		
Has dependents	52 (36.9)	41 (38.7)
Highest qualification, y of education (%)		
No qualification	11 (7.8)	4 (3.8)
General certificate of secondary education	35 (24.8)	25 (23.6)
A level	25 (17.7)	16 (15.1)
National vocational qualification	26 (18.4)	17 (16.0)
Higher national certificate/diploma	16 (11.4)	7 (6.6)
Degree	18 (12.8)	27 (25.5)
Higher degree	9 (6.4)	9 (8.5)
Other	1 (0.7)	1 (0.9)
Years of education (SD)	14.2 (3.8)	14.4 (2.8)
Previous treatment		
Physiotherapy	69 (49.6)^a^	42 (40.4)^a^
Psychology	25 (18.0)^a^	17 (16.4)^a^
Occupational therapy	22 (15.8)^a^	8 (7.7)^a^
Specialist inpatient rehabilitation	5 (3.7)^a^	4 (3.9)^a^
Symptom duration, y		
Mean (SD)	5.2 (7.2)	4.4 (4.9)
Dominant motor symptom (%)		
Weakness	47 (33.3)	31 (29.2)
Gait disturbance	45 (31.9)	35 (33.0)
Tremor	21 (14.9)	13 (12.3)
Mixed movement disorder	19 (13.5)	16 (15.1)
Jerks	7 (5.0)	6 (5.7)
Dystonia/fixed dystonia	2 (1.4)	5 (4.7)
Body part affected, dominant hand^b^ (%)		
Left upper limb	68 (48.3)	43 (40.6)
Right upper limb	68 (48.3)	45 (42.5)
Left lower limb	99 (70.2)	74 (69.8)
Right lower limb	92 (65.3)	75 (70.8)
Head/neck	36 (25.5)	20 (18.8)
Trunk	31 (22.0)	13 (12.3)
Dominant hand, right	128 (90.8)	97 (91.5)

a Variables with missing data, so the denominator is less than 141 for specialist physiotherapy or 106 for treatment as usual.

b Multiple sites/body parts could be affected.

### Cost of the Intervention

The mean number of treatment sessions received in the specialist physiotherapy group for the 141 participants included in the analysis was 8.62 (SD 2.40) for a total cost per participant of £457 (SD 72) for physiotherapy sessions only. 82% of participants (n = 87) in TAU received community neurologic physiotherapy. The mean number of sessions of physiotherapy received was 5.13 sessions (SD 7.05, mean of 6.25 (SD 7.32) when participants who received no sessions are excluded) for a total mean cost per participant of £272 (SD 374, £331 (SD 388) when participants who received no sessions are excluded).

Physiotherapy training was delivered over 5 days at a total cost of £33,788 for the 17 therapists trained or £1,988 per therapist. Because 179 participants in the specialist physiotherapy arm received the intervention, this translates to an (upper) conservative estimate of £189 per participant, given that therapists are likely to deliver the intervention to a greater number of patients than in usual practice. The mean cost per participant for specialist physiotherapy plus training was £646 (SD 72) compared with £272 (SD 374) in TAU (no training costs included).

### Resource Use and Costs

Descriptive statistics for resource use, presenteeism, and absenteeism are reported in eTables 2 and 3.

Table 2 summarizes the mean complete case costs and mean adjusted cost difference for health and social care and wider societal costs. There were no significant differences between specialist physiotherapy and TAU for any cost category. From a health and social care perspective, the specialist physiotherapy group costs £208 (95% CI £–1,410 to £994) less than TAU at 12 months, adjusting for baseline, site, and physiotherapist as a random effect. From a wider societal cost perspective, specialist physiotherapy costs £5,519 (95% CI –£15,460 to £4,423) less than TAU when excluding welfare costs and £5,438 (95% CI –£15,106 to £4,229) less when including welfare costs.

**Table 2:** Mean Cost per Participant (2021/2022 British Pounds)

	Specialist physiotherapy		Treatment as usual		Adjusted difference specialist physiotherapy minus TAU^a^ (95% CI)
	N	Mean (SD)	N	Mean (SD)	
Community service cost					
Baseline	141	347 (366)	106	278 (398)	
6 mo	131	244 (305)	96	306 (376)	
12 mo	134	181 (193)	95	239 (338)	
Total at 12	128	418 (429)	90	539 (631)	−154 (−289 to −18)
Specialist medical cost					
Baseline	141	509 (379)	106	577 (560)	
6 mo	131	381 (488)	96	312 (352)	
12 mo	134	289 (478)	95	216 (367)	
total at 12	128	679 (818)	90	516 (623)	167 (−22 to 357.00)
Inpatient and A and E					
Baseline	141	1,345 (2095)	106	951 (1,560)	
6 mo	131	811 (1787)	96	776 (1,548)	
12 mo	134	574 (1,504)	95	722 (1,504)	
Total at 12	128	1,265 (2,140)	90	1,494 (2,597)	−441 (−1,036 to 155)
Adaptations and wheelchairs					
Baseline	141	327 (863)	106	616 (4,139)	
6 mo	131	307 (982)	96	192 (453)	
12 mo	134	155 (639)	95	267 (1,053)	
Total at 12	128	470 (1,400)	90	478 (1,401)	10 (−366 to 386)
Caring, help, and transport—state funded					
Baseline	141	102 (416)	106	34 (180)	
6 mo	131	85 (379)	96	48 (207)	
12 mo	134	109 (510)	95	17 (79)	
Total at 12	128	192 (821)	90	56 (215)	44 (−40 to 129)
Medications					
Baseline	141	75 (154)	106	76 (132)	
6 mo	131	104 (212)	96	104 (245)	
12 mo	134	85 (204)	95	117 (326)	
Total at 12	128	188 (407)	90	230 (517)	−39 (−163 to 84)
Total health and social care resource use cost					
Baseline	141	2,706 (2,994)	106	2,532 (4,522)	
6 mo	131	1932 (2,616)	96	1739 (2097)	
12 mo	134	1,394 (1996)	95	1,579 (2,496)	
Total at 12	128	3,214 (3,581)	90	3,314 (4,279)	−208 (−1,410 to 994)
Private health care					
Baseline	141	106 (423)	106	99 (241)	
6 mo	131	110 (371)	96	112 (233)	
12 mo	134	79 (363)	95	92 (191)	
Total at 12	128	193 (566)	90	209 (365)	−19 (−141 to 103)
Paid carers—OOP					
Baseline	141	632 (3,482)	106	623 (2,761)	
6 mo	131	449 (2,270)	96	1,000 (3,700)	
12 mo	134	395 (2,693)	95	645 (2,956)	
Total at 12	128	854 (3,671)	90	1,495 (5,805)	−721 (−1815 to 374)
Family and close others—time spent caring					
Baseline	141	9557 (17,815)	106	9939 (19,623)	
6 mo	131	8,651 (18,035)	96	8,426 (17,649)	
12 mo	134	6,421 (14,792)	95	9417 (22,279)	
Total at 12	128	15,206 (27,574)	90	17,867 (38,213)	−2,668 (−10662 to 5,327.07)
Family and close others—time off work					
Baseline	141	659 (2,314)	106	843 (3,353)	
6 mo	131	504 (2,210)	96	614 (3,952)	
12 mo	134	278 (893)	95	180 (526)	
Total at 12	128	754 (2,498)	90	833 (4,120)	−48 (−959 to 864)
Transport—OOP					
Baseline	141	151 (482)	106	177 (453)	
6 mo	131	100 (235)	96	132 (420)	
12 mo	134	72 (239)	95	103 (295)	
Total at 12	128	177 (371)	90	243 (608)	−50 (−174 to 74)
Adaptations—OOP					
Baseline	141	385 (1,053)	106	637 (3,729)	
6 mo	131	420 (994)	96	300 (763)	
12 mo	134	188 (550)	95	270 (924)	
Total at 12	128	536 (1,101)	90	561 (1,299)	−13 (−327 to 301)
Total wider cost					
Baseline	141	13,538 (19,500)	106	14,008 (21,961)	
6 mo	131	11,661 (18,972)	96	11,709 (18,510)	
12 mo	134	8,550 (15,610)	95	12,106 (23,495)	
Total at 12	128	20,180 (28,972)	90	23,689 (40,153)	−3,265 (−11578 to 5,048)
Total productivity loss					
Baseline	141	5,493 (13,353)	106	4,594 (8,823)	
6 mo	132	1962 (6,661)	97	2,624 (7,118)	
12 mo	135	2,239 (8,121)	95	2,189 (5,207)	
Total at 12	129	4,351 (13,650)	91	5,006 (10,652)	−1,112 (−4,099 to 1876)
Total wider cost including productivity losses					
Baseline	141	19,031 (23,442)	106	18,602 (23,465)	
6 mo	131	13,638 (20,871)	96	14,361 (21,857)	
12 mo	134	10,806 (17,118)	95	14,295 (24,824)	
Total at 12	128	24,565 (32,686)	90	28,751 (44,311)	−5,519 (−15460 to 4,423)
Welfare payments					
Baseline	141	3,784 (3,205)	106	3,074 (3,045)	
6 mo	131	4,209 (3,525)	96	3,922 (3,611)	
12 mo	134	4,555 (3,769)	95	4,033 (3,661)	
Total at 12	128	8,788 (6,940)	90	7,728 (6,879)	−440 (−1,676 to 796)
Total wider cost productivity and welfare					
Baseline	141	22,815 (23,566)	106	21,676 (24,059)	
6 mo	131	17,847 (21,331)	96	18,282 (22,321)	
12 mo	134	15,361 (18,147)	95	18,328 (25,741)	
Total at 12	128	33,353 (34,316)	90	36,479 (45,678)	−5,438 (−15106 to 4,229)

Abbreviations: A and E = accident and emergency; OOP = out of pocket.

a Adjusted for site, controlling for baseline, and with a random effect for physiotherapist.

When the cost of the intervention (including training) is added, the adjusted mean health and social care cost per participant for specialist physiotherapy was £3,814 (95% CI £3,194–£4,433) compared with £3,670 (95% CI £2,931–£4,410) for TAU, with a mean incremental cost of £143 (95% CI £-825–£1,112) compared with TAU.

### Outcomes

Using the VSE to calculate health state utilities from the EQ-5D-5L (a preference-based measure of health-related quality of life where 1 is perfect health and death is anchored at 0), specialist physiotherapy resulted in a significantly higher utility of 0.054 (95% CI 0.002–0.107) at 6 months compared with TAU and when adjusting for baseline. The difference was not significant at 12 months (0.040, 95% CI –0.014 to 0.095). There was no significant difference in QALYs over the 12-month duration of the trial (0.030, 95% CI –0.007 to 0.067) (Table 3).

**Table 3:** Mean EQ-5D-5L Utilities and QALYs

EQ-5D-5L—Value set for England, mean (SD)	Specialist physiotherapy		Treatment as usual		Adjusted difference^a^ (95% CI)
	n	Mean (SD)	n	Mean (SD)	
Baseline	141	0.424 (0.290)	106	0.462 (0.264)	
6-mo	134	0.492 (0.291)	97	0.452 (0.294)	**0.054 (0.002 to 0.107)** ^a^
12-mo	137	0.483 (0.324)	97	0.480 (0.270)	0.040 (−0.014 to 0.095)
QALYs	133	0.475 (0.264)	93	0.476 (0.234)	0.030 (−0.007 to 0.067)
EQ-5D-5L—5L to 3L mapping, mean (SD)					
Baseline	141	0.310 (0.296)	106	0.354 (0.286)	
6-mo	134	0.383 (0.311)	97	0.341 (0.310)	0.069 (−0.0004 to 0.139)
12-mo	137	0.370 (0.342)	97	0.368 (0.298)	0.049 (−0.020 to 0.117)
QALYs	133	0.366 (0.281)	93	0.365 (0.254)	0.042 (−0.004 to 0.073)

Abbreviation: QALY = quality-adjusted life year.

a Adjusted for site, controlling for baseline, and with a random effect for physiotherapist.

### Cost-Effectiveness Analysis

**Figure 1:**
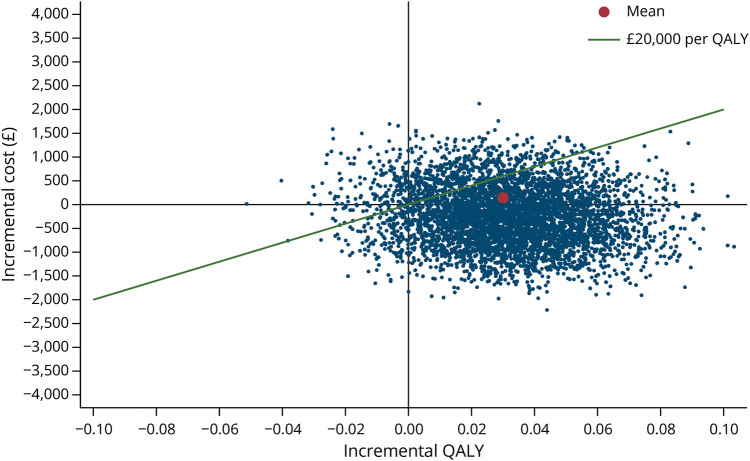
Cost-Effectiveness Plane From a Health and Social Care Perspective Based on VSE in EQ-5D-5L VSE = value set for England.

**Figure 2:**
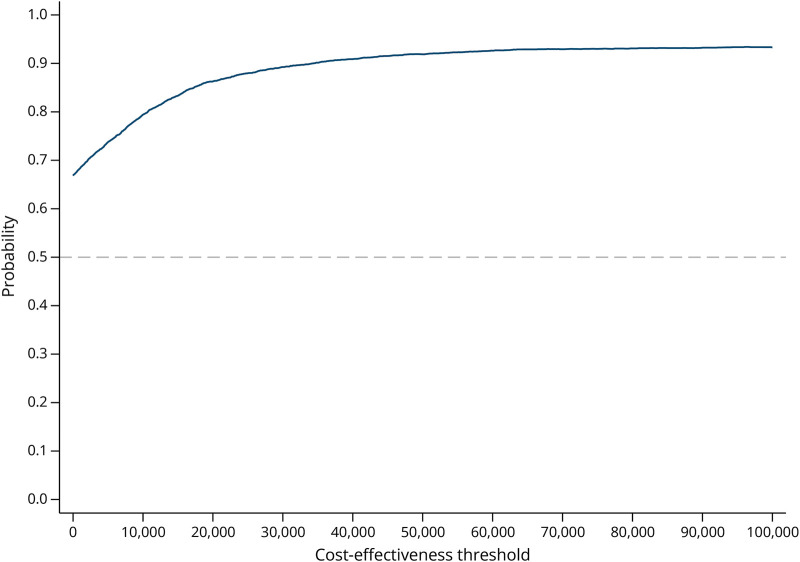
Cost-Effectiveness Acceptability Curve From a Health and Social Care Perspective Based on VSE in EQ-5D-5L VSE = value set for England.

The mean incremental cost per QALY gained for specialist physiotherapy compared with TAU from a health and social care perspective was £4,133 (mean incremental cost of £143 [95% CI £–1,080 to £1,367] divided by mean incremental QALYs of 0.03 [95% CI –0.01 to 0.08]). The cost-effectiveness plane is illustrated in Figure 1, showing that 63% of bootstrap iterations fall in the southeast quadrant, where specialist physiotherapy costs less and results in more QALYs than the intervention. The CEAC is presented in Figure 2. At the £20,000 threshold, the threshold commonly used by National Institute for Health and Care Excellence in England and Wales to determine whether an intervention is cost-effective compared with TAU,^26^ the probability of cost-effectiveness was 86% from the health care cost perspective. For the wider societal costs, the specialist physiotherapy dominates (lower cost and greater QALY mean point estimates) with an 89% probability that it is cost-effective at a £20,000 QALY threshold (eTable 4 and eFigures 2 and 3).

### HES

Descriptive statistics for HES from NHS England compared with self-reported response to the CSRI for 116 participants are reported in eTable 5. The mean cost per patient over 12 months for inpatient costs using data from NHS England was twice that of using patient-reported responses in the CSRI. This is due to the two patients who had long inpatient stays in HES missing self-reported data at 6-month and 12-month follow-up and hence not being included in the analysis of the CSRI. Overall, the proportion of participants reporting a hospital inpatient stay was lower in the CSRI (21/101, 21%) than in HES (54/116, 53%).

The difference between the two groups for A and E and inpatient stays was £84 greater (95% CI –£1,409 to £1,577) for specialist physiotherapy compared with TAU using data from NHS England and £484 less (95% CI –1,321 to 353) for the same patient cohort in the patient-reported population. The difference in the total cost of outpatient contacts with specialists was £165 greater (95% CI –£278 to £609) for specialist physiotherapy compared with TAU in the NHS England data set and £2 less (95% CI –£334 to £330) in the self-reported data.

If the difference in costs using data from NHS England (£84) is added to the additional cost of specialist physiotherapy (£374) and divided by the incremental QALYs (0.03), the incremental cost-effectiveness ratio would be £15,267 per QALY gained.

### COVID-19

The results for the COVID-19 subgroup analyses are reported in eTable 4. Participants randomized after the pandemic had a higher probability of specialist physiotherapy being cost-effective (eTable 4).

## Discussion

The results of this study represent the first economic evaluation alongside a powered randomized controlled trial of physical rehabilitation for FMD. It provides important evidence on the significant resource use implications of FMD and its detriment to health-related quality of life. The complex nature of FMD makes conducting research in this area challenging, with this trial providing some learning on how this could be improved in future studies. Although we found no significant differences in costs or QALYs between specialist physiotherapy and TAU, there is a high probability that it is cost-effective from a health and social care perspective and when wider societal costs are included. The ongoing cost of training physiotherapists is also likely to be lower after initial implementation, further increasing the probability that the intervention is cost-effective.

This is one of the first trials we are aware of that comprehensively captures the wider societal cost for people with FMD. Help from family and close others makes up a significant proportion of the total wider societal costs, with a total cost of £15,206 over 12 months for people who received specialist physiotherapy and £17,867 over 12 months for people who received TAU. This is similar to the cost of family and close others providing unpaid support in dementia, which has been estimated at approximately £16,032 a year.^27^ There is an issue though when capturing productivity losses for this group of patients. Productivity losses can only be measured for people who are actively employed. Because only 45% of participants were working at baseline, productivity losses were experienced by less than half of the study participants. This must be considered in future trials because it can raise equity issues, where people in employment are valued more highly than those who are not.

Our findings need to be interpreted alongside the findings in the clinical trial article, which found no significant difference on the primary outcome of the SF36 Physical Functioning domain.^10^ However, the specialist physiotherapy group was twice as likely to report an improvement in their motor symptoms at 12 months on a patient-reported Clinical Global Impression Scale, and specialist physiotherapy also led to significantly higher scores on SF36 Physical Role Limitations, SF36 Social Functioning, Hospital Anxiety and Depression Scale Anxiety,^10^ and EQ-5D-5L at 6 months, as reported in this article.

The contrasting findings between the economic evaluation and analysis of the primary outcome of the trial may in part be a result of a different burden of proof for economic evaluations to evaluate cost-effectiveness compared with determining clinical effectiveness. The outcomes of interest in economic evaluations, including resource use, costs, and QALYs, are not powered for as the primary outcome. Furthermore, the skewed nature of the data and difficulty with calculating 95% CIs for the incremental cost effectiveness ratio indicate that economic evaluations do not use *p* values to determine cost-effectiveness but, instead, present the probability that the intervention is cost-effective to inform decision making.^28^ Another important contributor to the different findings is likely to be that the primary outcome of clinical effectiveness was an overly narrow view of the potential benefits of physiotherapy. The secondary outcome, the patient-reported Clinical Global Impression Improvement score, allows for a broader assessment of potential impacts that specialist physiotherapy can have across various aspects of patients' lives, including the cost impact, thus capturing a wider range of outcomes. It is notable that consensus recommendations for outcome measures in FND (published after this trial was planned) have recommended the patient-reported Clinical Global Impression of Improvement as the primary outcome measure in trials of interventions in FND.^29^

We are not aware of any other studies that have evaluated the reliability of self-completed resource use questionnaires compared to medical records for people with FMD. Both methods of data collection had strengths and weaknesses, with NHS England routine data being more objective and less prone to loss to follow-up or recall bias and self-reported questionnaires able to capture a wider array of costs beyond secondary hospital care. In addition to limitations to NHS England data noted elsewhere in the literature,^30^ it did not cover all sites and there were some data-recording issues for 1 site that meant that we could not use the data. We were unable to use equivalent data for Scotland (obtained from the Information Services Division) because it does not contain sufficient information to conduct costings. Similar to the findings of a systematic review,^3^ costs varied by data collection method, with the total mean cost per patient calculated from the self-reported data being half that of the routine data. The mean patient-level differences between specialized physiotherapy and TAU were more comparable, suggesting that the differences between self-reported and medical record data were not related to the intervention but something else. Research going forward might want to consider a hybrid approach to resource use data collection, where health care resource use is collected from patient files, but still including some self-completed questionnaires to capture wider costs, given their importance.

The intervention resulted in greater cost savings and QALY benefit for participants recruited after COVID-19 restrictions had been lifted, suggesting a difference in effect at different times during the pandemic. This may reflect improvement with practice in the skills of the physiotherapists providing specialist physiotherapy because those randomized to TAU after lockdown restrictions had a similar amount of physiotherapy to participants treated before lockdown. It may also reflect the overwhelming impact of the COVID-19 pandemic on health-related quality of life and access to health care services, given that participants in Group B, those who were recruited and could finish treatment before the COVID-19 pandemic but were followed up during the early stages of the pandemic, made up 84% of Groups A and B.

Finally, economic evaluations should consider all costs and consequences relevant to the new intervention being assessed.^31^ It is possible that the benefits and costs of the Physio4FMD intervention extend beyond the follow-up period of the trial and hence lifetime cost-effectiveness might be different to that reported here. Given the limited evidence available in the literature on specialist physiotherapy, costs, or QALYs for people with FMD, it was not possible to extrapolate the findings beyond the time horizon of the trial.

In conclusion, the specialist physiotherapy protocol for FMD has a high probability of being cost-effective compared with (non-FMD specialist) neurologic physiotherapy. These results need to be interpreted alongside the clinical outcomes reported elsewhere, including the finding that the primary outcome was not statistically significant but participants in specialist physiotherapy were twice as likely to report improvement in their motor symptoms and they reported very high levels of satisfaction with treatment.
